# Correlation of Serum Vitamin D Levels with Chronic Rhinosinusitis Disease Severity

**DOI:** 10.22038/ijorl.2019.31926.2050

**Published:** 2020-01

**Authors:** Vahid Zand, Mohammadhossein Baradaranfar, Sedighe Vaziribozorg, Mohammad Mandegari, Mahzad Mansourimanesh, Nasir Saeidieslami

**Affiliations:** 1 *Department of Otolaryngology - Head and Neck Surgery* *, Otorhinolaryngology Research Center, Shahid Sadoughi University of Medical Sciences, Yazd, Iran.*; 2 *Department of Anesthesiology* *, Shahid Sadoughi University of Medical Sciences, Yazd, Iran.*

**Keywords:** CRS w NP, Lund-Mackay Scale, Vitamin D, SNOT-22

## Abstract

**Introduction:**

The present study was conducted to investigate the association between the serum vitamin D levels and severity of disease in chronic rhino sinusitis (CRS) patients.

**Materials and Methods:**

This prospective cross-sectional study was conducted on a total of 93 patients suffering from chronic rhino sinusitis with nasal polyposis (CRS w NP). Serum level of 25-hydroxyvitamin D was detected using a simple blood test. A22-item questionnaire, namely the sinonasal outcome test-22 (SNOT-22), was used to assess the subjective disease severity and patients' quality of life. In addition, the radiographic signs of the disease severity were evaluated using the Lund-Mackay Scale (LMS).

**Results:**

The mean age and serum vitamin D level of the patients were measured at 37.7±13.6 years and 24.6±16.9 ng/ml, respectively. Moreover, the mean of LMS and SNOT-22 scores were calculated at 14.2±11.2 and 40.8±17.6, respectively. There was a negative correlation between the SNOT-22 and serum levels of vitamin D (P=0.034). Similarly, LMS and serum vitamin D levels were correlated negatively (P=0.027). Furthermore, the results revealed a direct relationship between LMS and SNOT-22 (P<0.0001).

**Conclusion:**

According to the obtained results, there was a significant relationship between the serum vitamin D levels and severity of disease in patients with CRS w NP. Therefore, serum vitamin D levels could be added to the routine workup of the patients suffering from CRS w NP.

## Introduction

Rhino sinusitis is a group of diseases associated with the inflammation of the nasal mucosa and paranasal sinuses. When the symptoms last for at least 12 consecutive weeks, it is called chronic rhino sinusitis (CRS). TheCRS can be associated with nasal polyposis (CRSwNP) or without nasal polyposis (CRSsNP). The CRSsNP is a persistent inflammation resulting from the lack of the complete remission of acute infectious rhino sinusitis. On the other hand, CRSwNP is a disorder with various causes and pathogenesis, including fungi, resistant bacteria, super antigens, biofilms, mucosal disorders, environmental stimuli, sinonasal obstructions (especially osteomeatal complexes), and osteitises (1,2).

Vitamin D deficiency is related to atopic diseases, such as asthma, allergic rhinitis, and anaphylaxis. In addition, it is known that it has a deleterious effect on the musculoskeletal system (3,4). Currently, many physicians measure the level of vitamin D as part of a preliminary laboratory test. From the immunological viewpoint, CRS w NP results from T helper II activity, while CRSsNP is a T helper type I driven inflammation (5).

Some signs and symptoms of CRSwNP result from type 2 immune response to a variety of stimulants.

 Vitamin D can inhibit the synthesis and release of *interleukin 4* (IL-4) andIL-10. The IL-4 and IL-10 are the most prominent cytokines in type 2 immune response, which provoke synthesis and secretion of interferon gamma, as the most important cytokine in type 1 immune response (6,7). Moreover, vitamin D may contribute to the effects of glucocorticoid on cells, and higher doses of glucocorticoids are needed in vitamin D deficiency states to achieve therapeutic effects (8,9). Recently, Vitamin D gained attention in medical research and its role in the pathophysiology of several chronic diseases has been investigated (10-12).

Since CRS is associated with asthma and atopy, it can be assumed that vitamin D in CRS plays a similar role to that in asthma and atopy. With this background in mind, the current study was conducted to investigate the association between serum vitamin D levels and severity of disease in CRS patients. 

## Materials and Methods

The present study was approved by the Local Ethics Committee and written informed consent was obtained from all subjects. A total of 93 patients within the age range of 18-65 years with CRS w NP who referred to the ear, nose, and throat clinics of Yazd, Iran, were selected in this cross-sectional study, within 2016-2017. The diagnosis of CRS w NP was based on the criteria described by The European Position Paper on Rhino sinusitis and Nasal Polyps (2012) (13). The exclusion criteria were use of systemic steroids and vitamin D supplements one year prior to the study and affliction with such illnesses as chronic kidney disease, chronic liver disease, granulomatous disease, and sarcoidosis. Demographic and clinical information, such as gender, age, weight, height, *body mass index* (BMI), past medical history, surgical and family history, symptom duration, smoking status (Yes/No), and allergy, were collected using a questionnaire. The 25-hydroxyvitamin D was measured using the Euro immune commercial kit. This kit was designed based on the enzyme-linked immunosorbent *assay* method for the determination of 25-hydroxyvitamin D in a serum or plasma sample. The patients were also asked to fill out the sinonasal outcome test-22 (SNOT-22) questionnaire after providing them with the required instruction regarding the questionnaire completion. However, this instrument was completed by an otolaryngology resident in some cases. A recent review study investigating 15 specific questionnaires for patients with CRS, based on reliability, efficiency, and ease of use concluded that the SNOT-22 questionnaire was the most appropriate tool to assess the subjective severity of disease and quality of life in patients with CRS (14).The computed tomography scans of paranasal sinuses (PNS-CT) obtained from the patients were also evaluated and scored according to the Lund-Mackay Scale (LMS) by another otolaryngology resident, blinded to all other data. The relationship between serum vitamin D levels and the scores obtained from SNOT-22 and LMS were evaluated by the Pearson correlation analysis for statistical significance. in R software (15). A p-value less than 0.05 was considered statistically significant.

## Results

Out of 93 patients in the study,55 participants were male. The mean age of the participants was measured at 37.7±13.6 years (age range: 18-65 years). In addition, the mean level of serum vitamin D was estimated at 24.6±16.9 ng/ml. This value was obtained as 23.3±11.5 and 23.5±19.1 ng/ml in males and females, respectively, which was not significantly different. Weight, height, BMI, and age of the patients showed no significant correlation with the mean serum vitamin D level, LMS score, and SNOT-22 score. 

**Fig1:**
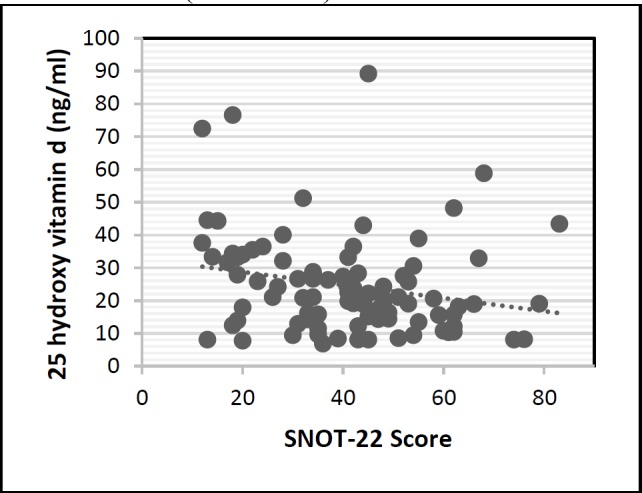
The relationship between SNOT-22 score and serum vitamin D levels

There were no significant differences in means of serum vitamin D level, LMS and SNOT-22 scores between patients regarding their smoking habits (P=0.272) and allergy status (P=0.340). Furthermore, the mean LMS score was calculated at 14.2±11.2, which was 14.4±5.7 in men and 14.8±8.1 in women. There was no significant difference between men and women in terms of mean LMS score (P=0.771).Moreover, the means NOT score was measured at 40.8±17.6. This value was estimated at 37.9±14.2 and 44.9±19 in men and women, respectively. The results also revealed a significant difference between males and females regarding the mean SNO T scores (P=0.046). The Pearson correlation coefficient between the SNOT-22 and serum levels of vitamin D was -0.22 (P=0.034). Results of serum vitamin D levels and the score obtained from SNOT for each patient are plotted in (Fig.1). A contrary correlation was also observed between LMS and serum levels of vitamin D (P=0.0271).

There was an inverse correlation between the SNOT22 and serum levels of vitamin D (P <0.035).

**Fig 2:**
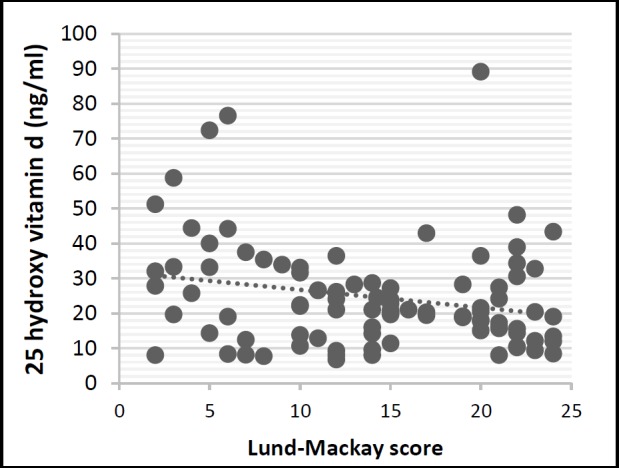
Relationship between the Lund-Mackay score and the serum vitamin D levels.

**Fig 3:**
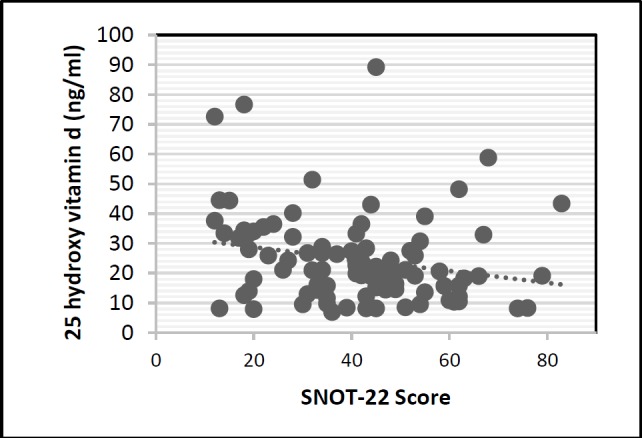
Correlation between Lund-Mackay and SNOT-22 scores

Figure 2 shows serum vitamin D levels and score obtained from the LMS for each patient. The association between the radiological signs and clinical symptoms of the disease was studied. Pearson correlation coefficient between LMS and SNOT-22scores was measured at 0.41(P<0.0001).The scores obtained from the SNOT and LMS for each patient is depicted in (Fig.3).

Serum vitamin D levels inversely correlate with Lund-Mackay scores. Lower vitamin D levels correlated with higher radiologic disease severity as measured by Lund-Mackay scale (r=−0.23, P<0.05). 

Lund-Mackay scores correlate directly with SNOT-22 scores. Higher Lund-Mackay scores correlated with higher subjective symptoms severity as measured by SNOT-22 (r = 0.41, P< 0.05). 

## Discussion

While the cause and pathogenesis of acute rhinosinusitis is are typically associated with an infection, CRS results from complex inflammatory processes. Generally, CRS is a consequence of a vicious interaction between the host immune system and microbiome at the nasal and sinus mucosal surfaces. The immune barrier hypothesis is the main accepted host-driven theory to clarify the pathogenesis of CRS. Local defects in the mechanical and innate immune barriers increase the formation of microbial colonies and subsequent immune system responses (2).

Several studies have addressed the role of vitamin D deficiency in defective mechanical and immune barriers in inflammatory bowel disease and psoriasis (16,17). The role of vitamin D deficiency in the failure of mechanical and immunological barriers has not been specifically addressed in rhino sinusitis. However, vitamin D deficiency might affect the pathogenesis of rhino sinusitis by weakening these barriers. The main purpose of this study was to investigate the association between serum vitamin D levels in CRS patients with the severity of disease.

According to the results of the recent studies, patients with lower levels of serum vitamin D subjectively experience more severe disease and poorer quality of life. Additionally, higher LMS is associated with lower levels of serum vitamin D. Moreover, there is a direct correlation between the severity of radiological signs and severity of clinical symptoms. In a study performed by Moradzadeh et al., mild, moderate and severe vitamin D deficiency levels in women were defined as 25-39.9, 12.5-24.9 nmol/L, and < 12.5 mol/L, respectively. Furthermore, regarding males, the serum vitamin D levels of 25-34.9and < 25 mol/L were indicative of mild and moderate/severe vitamin D deficiencies, respectively(18).

Based on this classification, 23.7% of women with rhino sinusitis, participating in our study, had mild deficiency, and 55.3% of them had moderate and severe vitamin D deficiency. Additionally, 21.8% and 65.5% of men with rhino sinusitis had mild deficiency and moderate/severe vitamin D deficiency, respectively. Only 12.7% of men and 21.1% of women had normal levels of vitamin D. In several studies, patients with CRSwNP were reported to have a lower level of serum vitamin D than those with CRSsNP (19,20). A significant inverse relationship between SNOT-22 and vitamin D serum levels was observed in our study. In a study by Wang et al., the level of vitamin D in a group of patients with CRSwNP in Taiwan was significantly lower than that of the patients with CRSsNP. This variable was also reported to be inversely associated with nasal polyp size (21).

In another study performed by Christensen et al., there was no significant relationship between the symptoms of the disease based on total nasal symptom score (TNSS) and serum vitamin D level. Never the less, TNSS was found to have a direct relationship with the expression of vitamin D receptor genes and 24-hydroxylase gene. They concluded that the loss of the localized adjustment of vitamin D in the sinonasal tissue during CRS may be independent of serum vitamin D levels and that the control of vitamin D may be impaired at different levels (22). 

In the aforementioned study, 10 patients with CRSwNP were examined, and perhaps their failure to find a relationship between the severity of the symptoms and serum level of vitamin D was due to their low sample size. The results of a study conducted by Schlosseron patients with CRSwNP demonstrated a negative correlation (r=-0.32) between the levels of vitamin D and LMS score. Moreover, they found more severe symptoms and mucosal disease in the CT scan of CRS patients with vitamin D deficiency (23). Mulligan et al. also observed a negative relationship (R2=-0.55) between serum vitamin D levels and bone erosion in the PNS-CT of patients with chronic and fungal rhino sinusitis (20). 

In a study carried out by Wang et al., there was no significant relationship between serum vitamin D and Lund-Mackay criteria. However, a negative relationship was found between polyp grade and serum levels of vitamin D. Accordingly, the authors suggested that the serum level of vitamin D could be added to the routine check of patients with CRS and this data could potentially help to determine the severity of the disease (21).

In a number of studies, there was no relationship between the severity of clinical symptoms and CT findings (24,25). These studies did not use SNOT-22 to assess the subjective severity of the disease. Greguriæ et al. observed no relationship between the overall score of SNOT-22 and the LMS scores in CRS patients. However, they found an association between the nasal symptoms of SNOT-22 and LMS scores in patients with CRSwNP and facial pain (26). In a study carried out by Dousti et al., there was a weak correlation between the severity of clinical symptoms and severity of nasal sinus involvement based on CT scans (r=0.148)(27).However, Jalali et al. reported a relatively strong correlation between LMS and SNOT-22 scores(r=0.74) (28).

The results of the majority of studies were indicative of a weak relationship between the CT findings and symptoms of the disease; however, both of these are important in the evaluation of the sinusitis. Some researchers investigated the role of vitamin D supplements in treating patients with CRS. Faruk et al. showed that a daily intake of 4,000 vitamin D units for 4 weeks significantly reduced the symptoms of chronic sinusitis (29). However, the symptoms in patients suffering from asthma and rhino sinusitis were not recovered with vitamin D for 12 weeks (30). 

It is worthy of note that this study did not address the phenotype of rhino sinusitis. A 12-week treatment may be inadequate for proving the usefulness of the vitamin D therapeutic effects. It is worth noting that based on the results of our study, no cause and effect relationship was found between the serum vitamin D level and severity of the symptoms or its radiological signs. Waldron et al. observed a reduction in the serum vitamin D levels after knee and pelvic surgery. Accordingly, they described vitamin D as a negative acute phase reactant and considered it as an unreliable biomarker after acute inflammations. In addition, they concluded that vitamin D deficiency may be due to chronic inflammatory diseases (31).

Cannell et al. performed a systematic review of the interventional studies focusing on the role of vitamin in inflammatory diseases. In 6 out of 7 reviewed studies, patients with severe inflammatory conditions, who initially had high inflammatory markers and low vitamin D levels, demonstrated modestly lower inflammatory markers after receiving vitamin D supplements. Therefore, they concluded that the improvement of vitamin D levels could at some points decrease the level of acute-phase reactants in severe inflammatory conditions. However, inflammation may interfere with the metabolism of vitamin D (32).

## Conclusion

The results of the present study revealed was a significant relationship between the serum level of vitamin D and severity of the disease in patients with CRSwNP. Regarding this, serum vitamin D levels could be added to the routine workup of patients suffering from CRSwNP.

## Recommendations

To confirm the role of vitamin D in CRS, there is a necessity to conduct prospective studies and randomized controlled clinical trials with larger sample size. Moreover, future studies are recommended to characterize the type of relationship (cause and effect) between serum vitamin D levels and the incidence, exacerbation, and protraction of rhino sinusitis. Additionally, it is suggested to investigate the role of vitamin D supplementation in the treatment or prevention of rhino sinusitis recurrences.
